# Effects of Tyrode's solution osmolarities and milk on bull sperm storage above zero temperatures

**Published:** 2011

**Authors:** Farid Barati, Ahmad Ali Papahn, Mahsa Afrough, Mohammad Barati

**Affiliations:** 1 Department of Clinical Sciences, Faculty of Veterinary Medicine, Shahid Chamran University of Ahvaz, Ahvaz, Iran.; 2 Department of Basic Sciences, Faculty of Veterinary Medicine, Shahid Chamran University of Ahvaz, Ahvaz, Iran.; 3 Department of Applied Chemistry, University of Tabriz, Tabriz, Iran.

**Keywords:** *Bull sperm*, *Tyrode's solution*, *Whole milk*, *Osmolarity*, *Glycerol*

## Abstract

**Background**: Control of the medium osmolarity and temperature during long or short time sperm manipulation is essential.

**Objective:** The objectives of the present study were to find the effects of different osmolarities of modified Tyrode's solution and milk on the bull sperm during incubation at above zero temperatures.

**Materials and Methods:** Semen samples were collected twice from five Najdi bull. Centrifuged and most parts of seminal plasma were removed. First experiment: The concentrated semen were splited into nine aliquots to incubate in three different osmolarities (200, 300 and 400 mOsm) at three temperatures (5, 25 and 39°C) for 15 and 60 min of incubation. Second experiment: The semen samples were splited, mixed with the same volume of whole cow milk (5 and 25°C) and milk with 7% glycerol (5°C) and incubated for 15 and 60 min.

**Results: **Sperm motility severely affected (p<0.05) by incubation at low ionic tension (200 mOsm/l) especially at low temperature (5°C). The impact of low osmolarity on sperm viability can reduce by increasing the incubation temperature to 39°C. The decreased sperm motility, which was induced by lowering osmolarity, was not improved (p>0.05) by increasing temperature during 1 h of incubation. Milk can protect the sperm viability and motility at cool conditions and there is no beneficial effect of glycerol in combination of milk on sperm incubation at above zero temperatures (p<0.05).

**Conclusion: **Iso- and hyper-osmotic solutions protect bull sperm motility and viability at 25 and 39°C, while milk can be used for protecting sperm at 5°C.

## Introduction

Sperm is exposed to different medium osmolarities and temperatures during freezing or manipulation. Cryoprotectants are high molecular weight substances like glycerol, ethylene glycol or di methyl sulfoxide which are cytotoxic at high temperatures. After exposing to the cryoprotectants at low temperatures, the intracellular water is excluded from the sperm and the osmolarity of the intracellular environment increased, this effect is also very dangerous for the sperm, which is called "solution effects" (1). 

Therefore, the cryopreservation protocol should be able to reduce these risk factors: lowering the temperature (from 38 to -196°C) and toxic effects of the cryoprotectants. Cooling rate, equilibration time and temperature are three important factors in sperm freezing programs. Impact of cooling rate on sperm motility may be mediated by changes in osmolarity (2). On the other hand, incubating the sperm at above zero temperatures for short time transportation for artificial insemination in some domestic animal species and it’s manipulation during human sperm processing have been considered. Effects of non-ionic and ionic diluents on sperm incubation at above zero temperatures have been investigated in different species (3-5). Some studies reported the morphologic abnormalities of sperm after exposing to permeating (6) and ionic-based (4) solutions at above zero temperatures. Liu and Foote (1997) have shown that the best bull sperm reaction for above zero temperatures incubation is depended on the medium osmolarity, temperature and time of incubation (7). Rigby *et al*, (2001) reported the beneficial effects of modified Tyrode’s solution for preserving the stallion sperm only when seminal plasma was to be removed (5). Increased DNA damage due to cooling bull sperm in skim-milk egg yolk at above zero temperature has been reported, too (8). These impacts of osmolarities on the sperm parameters may be influenced by the composition and temperature of medium (7), species (9), individuals (5, 10) and the sources of collected sperm (11). For many years, researchers have reduced the damages caused by freezing by the addition of natural protein and lipoprotein sources like egg yolk or milk in combination with the cryoprotectants (1). 

The beneficial effect of egg yolk for the preservation of cooled sperm has also been documented (7). There are reports of protecting effects of the milk based media on sperm motility during storage (8, 12). The aim of the present study was to evaluate the sperms reaction after exposing it to different osmolarities of Tyrode's solution and milk at different temperatures during two different incubation times.

## Materials and methods

**Bull semen**

Semen was provided from bulls (n=5) at the research center for Agriculture and Natural Resources of the Khuzestan province, Iran. Semen was collected using an artificial vagina (39°C) and transported to the laboratory in a light protected 35°C flask for 1 hour.

**Media preparation**

Modified Tyrode’s solution [in g/l, 8.18 NaCl, 0.24 KCl, 0.04 NaH_2_PO_4_, 2.18 NaHCO_3_, 0.08 MgCl_2_ 6H_2_0, 0.31 CaCl_2_ 2H_2_O, 0.94 glucose (all from Merk, Germany), and 2.48 HEPES 300 mOsm/l (Sigma, USA)] was the basic medium in experiment 1. The HEPES buffer was used to prevent the rapid changes in the medium pH. The modified Tyrode’s solution with 100 mOsm/l was prepared by diluting 1 part of Tyrode's with 2 parts of deionized water. To provide 500 mOsm/l solution of Tyrode’s, all values pf chemical components (without HEPES) of Tyrode's were multiplied by 1.66 and added to 1 liter of deionized water (7). 

Osmolarities of all solutions were measured and confirmed by a calibrated vapor pressure osmometer (Model 5500, Wescor, Logan, UT, USA). Whole cow milk (Choopan dairy industries, Iran) has been heated (to 95°C in boiling water over 30 to 45 min) to inactivate lactenin in the protein fraction because of its toxicity (13). The heated whole milk used alone or was supplemented with 7% glycerol (Merk, Germany) for the second experiment. All of the media in this study were supplemented with 0.25 mg/ml of gentamycin (Daru-pakhsh, Iran).

**Extension of semen**

Fresh semen samples were centrifuged at 500 g for 25 min to remove most of the seminal plasma to provide the sperm concentration of approximately 6 × 10^9^/ml. The concentrated semen was mixed 1:1 (v/v) with various media by gentle stirring, giving a final sperm concentration of approximately 3 × 10^9^/ml. As diluents and semen were combined in equal volumes, the final osmolarities of the suspensions of sperm were the means of the osmolarities of different media with that of semen (approximately 300 mOsm/l). All the procedures of semen extension were performed at the room temperature.

**Sperm analysis**

An expert person in this field of study undertook the sperm analysis procedures. The percentage of motile and viable sperm was estimated 15 to 60 min after initial exposure to the media. Before evaluation, samples were subjected to iso-osmotic modified Tyrode’s solution. A part of sperm was mixed with a part of Eosin B/Nigrosin stain (Sigma, USA) on a slide for 1 min and dried by placing it on a hotplate. A total of 100 sperm per sample was counted with the light microscopy at a magnification of 100. Unstained sperms were considered to be viable (14). Sperm progressive motility was estimated by placing a small volume of sperm suspensions, covered by a coverslip on a heat stage under a light microscopy.

**Experiment 1: **Sperm in Tyrode’s solution varying in osmolarity. The volume of sperm (n=5) in Tyrode’s solution was measured in a factorial experiment with three osmolarities of the Tyrode’s: semen combination (200, 300, and 400 mOsm/l), and examined after two different incubation times (15 and 60 min) at 5, 25 and 39°C and replicated with five semen samples from different bulls. The sperm suspension aliquots were placed in a water-jacketed microtube. Then the water-jacketed microtubes were placed in the refrigerator (5°C), room temperature (25°C) or a warm water bath (39°C).

**Experiments 2: **Sperm in milk with and without 7% glycerol Equal volumes of semen samples (n=5) were mixed with milk without glycerol at two temperatures (5 and 25°C) and with milk with 7% glycerol at 5°C. Progressive motility and viability of sperms were evaluated after 15 and 60 min of incubation. The experiment was replicated with five semen samples from different bulls.

**Statistical analysis**

In the first experiment, effects of osmolarity, temperature and duration of incubation and their interaction on the percentages of viable and progressive motile sperms, in a split plot design, were analyzed by ANOVA using General Linear Model (GLM) procedure of SAS (the main plot was the medium osmolarities and two subplots were the temperatures and the durations of incubation). In the second split plot design, the effects of diluents (milk with and without glycerol) through two times of incubation at different temperatures on the percentages of viable and progressive motile sperms were analyzed using GLM procedure of SAS. Least square means (LSmeans) were compared with pdiff test in SAS (15). Data are presented as the LSmeans ±standard error of means (SEM).

## Results

**Experiment 1**

**Figure 1:**
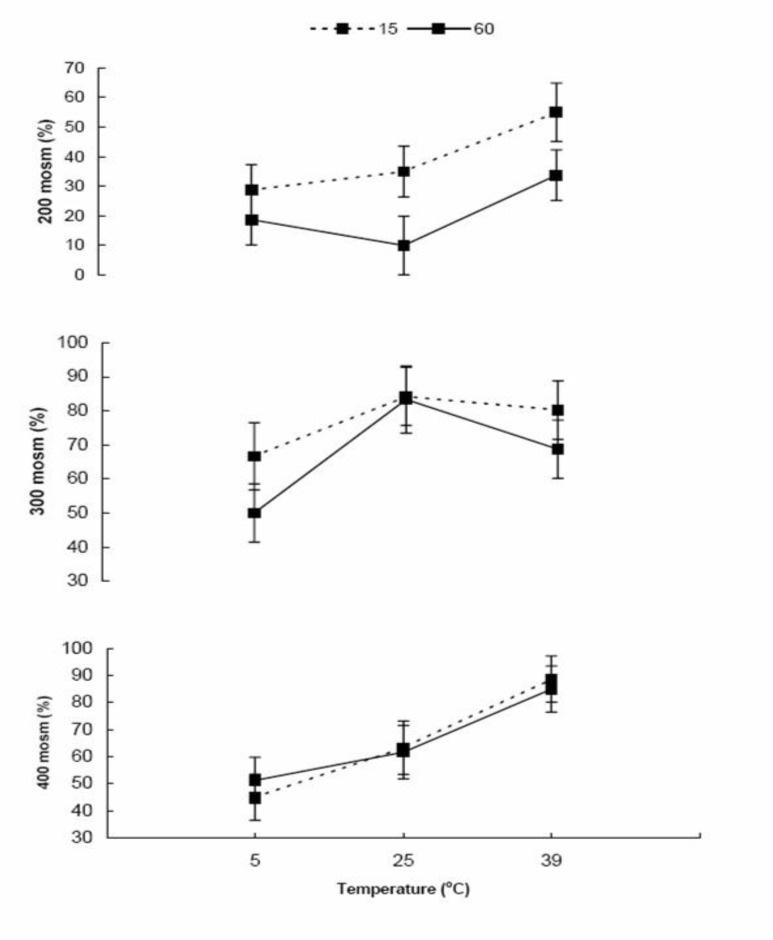
Bull sperm (n=5) viability after exposure to different osmolarities (200, 300 and 400 mOsm/l) at three different temperatures (5, 25 and 39°C) for two different incubation times (15 and 60 min).

**Figure 2:**
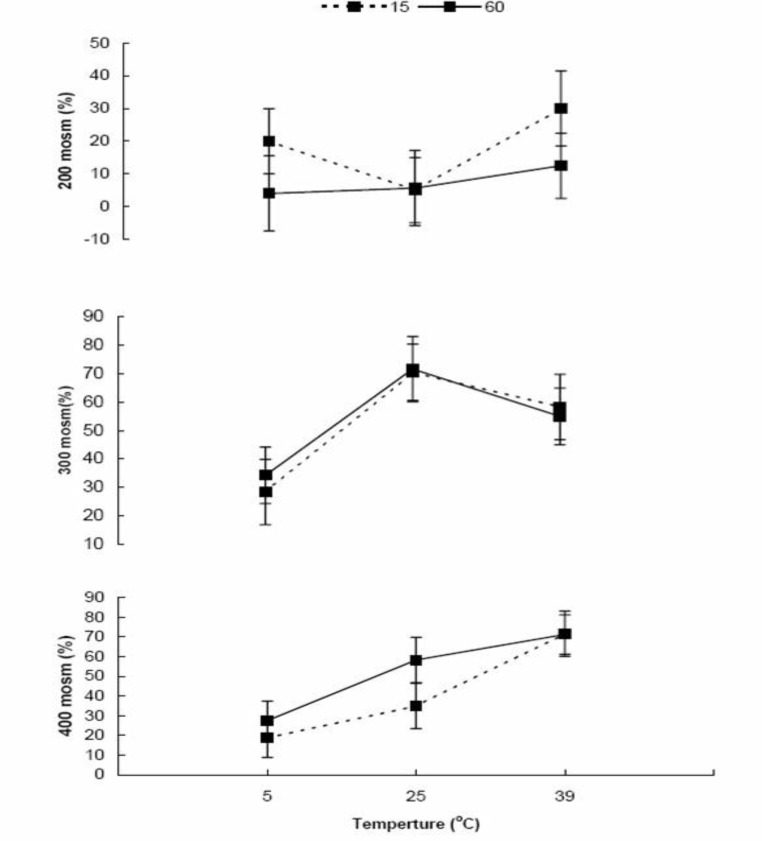
Bull sperm (n=5) progressive motility after exposure to different osmolarities (200, 300 and 400 mOsm/l) at three different temperatures (5, 25 and 39°C) for two different incubation times (15 and 60 min).

The results are presented in figures 1 and 2. Total sperm viability decreased after 60 min (51.4±3.02 %) compare to 15 min of incubation (60.8±3.01; p=0.032). The percentages of viable sperm were 43.4±3.6, 56.3±3.9 and 68.6±3.6 at 5, 25 and 39°C incubation, respectively (p<0.0001). Sperm viability significantly decreased (p=0.0001) by incubation in 200 mOsm (30.2±3.7 medium) compare to the 300 (72.2±3.7) and 400 (65.8±3.7) mOsm media. However, the percentage of viable sperms was not different between 300 and 400 mOsm (p=0.23) media. While there was not (p=0.81) any interaction between duration of incubation, temperature and medium osmolarity (Figure 1), a significant (p=0.04) interaction was observed between medium osmolarity and incubation temperature on sperm viability. Medium osmolarities of 300 and 400 mOsm/l, at 5°C, (53.8±6.5 *vs.* 48.1±6.05; p=0.25, respectively) significantly (p=0.0003) reduced the impact low osmolarity (200mOsm/l) on the percentage of viable sperms (23.7±6.05). The highest percentage of viable sperms, at 25°C, was observed (p=0.03) in 300 mOsm/l medium (83.8±6.5) compare to 200 and 400 mOsm/l media (22.5±6.5 *vs.* 62.5±6.9; respectively, p=0.0001). Although, incubation at 39°C (44.4±6.5%) reduced (p=0.01) the impact of 200 mOsm/l medium at 25 and 5°C on sperm viability, higher osmolarities (300 and 400 mOsm/l) are required (p=0.03) to optimize the percentage of viable sperm (74.5±6.05 *vs.* 86.9±6.05; p=0.15 respectively) at 39°C. The patterns of changes were similar at two different (15 and 30 min) durations of incubation (Figure 1).

There was no difference between 15 (37.5±3.6%) and 60 (37.8±3.6%) min of incubation on sperm progressive motility (p=0.95). Total sperm progressive motility (%) decreased (p=0.004) at 5°C (22.1±4.3) compare to 25 and 39°C (41±4.5 and 49.8±4.4 respectively; p=0.17). Overall sperm progressive motilities were 12.9±4.4, 53.01±4.4 and 47.1±4.4% at 200, 300 and 400 mOsm media, respectively (p=0.0001). While there was not (p=0.94) any interaction between duration of incubation, temperature and medium osmolarity (Figure 2), a significant (p=0.005) interaction was observed between medium osmolarity and incubation temperature on sperm motility (%). The highest (p=0.07) sperm motility at 5°C was observed in 300 mOsm/l (31.3±7.6%) compare to 200 and 400 mOsm/l solutions (12±7.6% *vs.* 23.1±7.4% respectively; p=0.3). The highest (p<0.03) sperm motility at 25°C was observed in 300 mOsm/l solution (71.1±7.6) compare to 200 and 400 mOsm/l (5.3±7.6 vs. 46.7±8.1 respectively; p=0.0006) solutions. The highest sperm motility (p=0.0001) at 39°C was in 400 mOsm/l (71.5±7.6) compare to 300 (56.7±7.6) and 200 (21.25±7.6) mOsm/l solutions. The patterns of changes were similar at two different (15 and 30 min) durations of incubation (Figure 2).

**Experiment 2**

The results of this experiment are presented in the tables I and II. There was no interaction between the duration of incubation, temperature and the composition of diluents on sperm viability (p=0.89) and progressive motility (p=0.45). Total percentages of live sperms were 67.6±6.3 and 52.2±6.3 after 15 and 60 min of incubation (p=0.1), respectively. Total sperm viability was not different (p=0.15) in milk at 25°C (70.5±7.8%), and 5°C (54.5±7.8%) and milk with glycerol at 5°C (54.6±7.8%). The patterns of sperm viability changes in different media were similar at two different incubation times (Table I; p=0.26).

Total sperm progressive motilities were 34.6±7.4% and 18.8±7.4% after 15 and 60 min of incubation, respectively (p=0.14). The percentage of progressive motile sperms was not significantly different (p=0.55) between milk at 25°C (32±9.1%), milk at 5°C (24.2±9.1%) and milk with glycerol at 5°C (23.7±9.1%). The patterns of sperm progressive motility changes in different media were similar at two different incubation times (Table II; p=0.77).

**Table I:** Effects of different media during two different incubation times (15 and 60 min) on the bull (n=5) sperm viability (LSmeans±SEM).

**Incubation time ** **(min)**	** Medium**		
	**Milk at 5°C**	**Milk at 25°C**	**Milk with glycerol at 5°C**
15	55±8.5 aA	85±8.5 bA	62.8±5.2 cA
60	54±8.5^ a^^A^	56±8.5 aB	46.4±5.2 aB

abc Values with different superscript within rows significantly differ (p<0.05).

AB Values with different superscript within columns significantly differ (p<0.05).

**Table II:** Effects of different media during two different incubation times (15 and 60 min) on the bull (n=5) sperm progressive motility (LSmeans±SEM).

**Incubation time ** **(min)**	** Medium**		
	**Milk at 5°C**	**Milk at 25°C**	**Milk with glycerol at 5°C**
15	29±9.3^ a^^A^	40±9.3 aA	34.6±9.3 aA
60	19.4±9.3 aA	24±9.3 aA	12.8±9.3 aB

a Values with different superscript within rows significantly differ (p<0.05).

AB Values with different superscript within columns significantly differ (p<0.05).

## Discussion

Incubation of sperm in an ionic base solution at low temperature decreased motility and viability of sperm especially under condition of lower ionic tension (200mOsm/l). Higher osmolarities increased sperm viability and motility in linear fashion thorough the temperature changes at least up to 1 hour of incubation. Data have shown that sperm motility increased significantly at 39°C, similar to the procedure for sperm capacitation during IVF (16). Using the Tyrode’s solution (5) and supplementation of storage media with modified Tyrode’s solution (17) were effective for sperm protection during liquid sperm storage. Liu and Foote (1997) have shown significant changes of sperm motility in the 200 and 400 mOsm/l Tyrode’s base solutions. When they adjusted the osmolarities by NaCl changes, they found many immotile sperms with intact plasma membrane at higher osmolarities (7).

The second experiment has shown the beneficial effects of whole milk base diluent for preservation of sperm at above zero temperatures especially at cool storage for at least 1 hour. Egg yolk and milk improved the output of sperm cryopreservation. Foote and Liu (1997) found that solution containing egg yolk (300 mOsm/l) protected the sperm motility during 1 hour of cool storage (7). Data of the present study also confirms that using whole milk can reduces the impact of lowering temperature on sperm viability and motility. The beneficial effect of milk is limited to the cool temperature and at the higher temperatures, it can not protect the sperm viability and motility. The mechanism by which milk protect the sperm surface is different from egg yolk, as protection appear to reside with the protein fraction rather than lipoprotein or phospholipids (13, 18). Batellier *et al* (2001) showed that while some milk fractions (ultrafiltrate, microfiltrate, and a-lactalbumin fraction) decreased spermatozoal survival, others (beta-lactoglobulin and native phosphocaseinate) were protective (19).

Comparing egg yolk and skim milk based extenders with the Ringer solution, Andrade *et al*, (2008) showed that the sperm motility was better preserved during the incubation from 30 to120 minutes for the semen diluted in yolk egg-citrate and skim milk-based extenders than from the Ringer extender (4). Sperm sensitivity to the osmotic changes was studied in many different species. Equine sperm is resistant to osmotic shock (9). There are individual variations in bull (10) and mouse (6) sperm resistance to osmotic shock. Source of the provided sperm may also have a significant effect on the osmotic behavior of sperm; epididymal sperm is more resistant than ejaculated sperm against osmotic stress (11). Impact of osmotic stress on sperm can be mediated thorough an oxidative stress (20). Cooling can also exert its effect on sperm thorough osmolarity changes (2). Zhang *et al* (2001) reported changes in sperm motility in a temperature-time-medium dependent relation. They found that glycin betaein can improve the sperm motility at lower temperatures, with no effect at 37°C (21). Zhou *et al* (2004) concluded that Poly Vinyl Alcohol (PVA) can be used to substitute for Bovine Serum Albumin (BSA) and 20°C is more suitable than 15°C for boar semen storage, and in vitro fertilizing capacity of spermatozoa is maintained for at least 8 days in Zorlesco+PVA at 20°C (22). Li *et al* (2006) showed that prefreezing treatment of bovine sperm with cholesterol-loaded cyclodextrin (CLC) increased the percentages of motile and viable sperms (23). The optimal conditions for preservation of mouse spermatozoa were 800 mOsmol KSOM containing 4 mg/ml BSA and a holding temperature of 4°C (24).

In conclusion, Iso- and hyper-osmotic solutions can protect the sperm motility and viability at 25 and 39°C, while non-ionic solutions (milk) can use for protection of sperm at 5°C incubation. 
